# Game Theory Meets Wireless Sensor Networks Security Requirements and Threats Mitigation: A Survey

**DOI:** 10.3390/s16071003

**Published:** 2016-06-29

**Authors:** Mohamed S. Abdalzaher, Karim Seddik, Maha Elsabrouty, Osamu Muta, Hiroshi Furukawa, Adel Abdel-Rahman

**Affiliations:** 1 Electronics and Communications Engineering Department, Egypt-Japan University of Science and Technology (E-JUST), Alexandria 21934, Egypt; maha.elsabrouty@ejust.edu.eg (M.E.); adel.bedair@ejust.edu.eg (A.A.-R.); 2 Kyushu University, Fukuoka 819-0395, Japan; muta@ait.kyushu-u.ac.jp (O.M.); furuhiro@ait.kyushu-u.ac.jp (H.F.); 3 Electronics and Communications Engineering Department, American University in Cairo, Cairo 11835, Egypt; kseddik@aucegypt.edu

**Keywords:** game theory, WSNs security, evolutionary game, game-theoretic based trust model, WSNs applications

## Abstract

We present a study of using game theory for protecting wireless sensor networks (WSNs) from selfish behavior or malicious nodes. Due to scalability, low complexity and disseminated nature of WSNs, malicious attacks can be modeled effectively using game theory. In this study, we survey the different game-theoretic defense strategies for WSNs. We present a taxonomy of the game theory approaches based on the nature of the attack, whether it is caused by an external attacker or it is the result of an internal node acting selfishly or maliciously. We also present a general trust model using game theory for decision making. We, finally, identify the significant role of evolutionary games for WSNs security against intelligent attacks; then, we list several prospect applications of game theory to enhance the data trustworthiness and node cooperation in different WSNs.

## 1. Introduction

Security and authentication in Wireless Sensor Networks (WSNs) face a more challenging environment compared to traditional networks. WSN has an ad-hoc nature in which the nodes can dynamically enter or leave the network, which leads to a variable network topology. Consequently, there is no predefined route for data replication. With the ambiguity of the nodes involved, a critical problem may occur when a malicious intruder attacks the system. In addition, power limitation can turn the node itself to behave selfishly in order to conserve its energy, which increases the risk of network malfunctioning. Therefore, the above-mentioned aspects of the WSNs make the security schemes in WSNs more challenging and vulnerable. For this reason, security in WSNs has gained increasing interest. Some techniques were developed to meet different WSNs security threats mitigation [1,2,3,4]. There are other techniques that are used for protection in WSNs that rely on the reputation principle [5,6,7,8,9,10,11,12,13,14,15,16,17,18]. These reputation-based techniques are out of the scope of this survey paper since our main focus in this paper is on game-theoretic-based protection techniques.

Game theory is a modern branch of intelligent optimization [19,20,21,22,23,24,25,26,27]. It tackles problems where cost functions of different entities are mutually dependent [19,22,23,24,25,26,27]. Since the rise of game theory in the late 1940s, it has been widely applied to model the behavior in a variety of applications. In [28], the authors discuss the promising features of game theory approaches for the wireless networks, while in [29,30], different trends of using game theory for WSNs have been reviewed. Recently, with the emergence of infrastructure-less and distributed systems, game theory has found its way in decentralized communication systems [31]. One of the problems in this category is related to security in WSNs. The security situation, which involves an interaction between the defender(s) and attacker(s), can be directly mapped to a game among players in which each player strives to promote its benefit. More particularly, having the action of the attacker(s) or the defender(s) depending on the counter-action of the other party places game theory as a perfect fit for this security model [22,24,25,26,32].

In this paper, we introduce a brief interpretation of the different game techniques presented in the literature to address WSN security. In addition, an overall view of the desired WSN properties in terms of security fulfillment is presented. We study the game theory based approaches for mitigating different WSN security threats from the state-of-the-art literature on the topic. We classify those approaches into two main categories, namely, cooperative games and non-cooperative games, and each summarizes the involved defense strategies based on game theory. Then, we propose a taxonomy of game theoretic defense strategies taking into consideration the attacked layer, attack features, attack consequences, convenient defense game approach, and game type. Afterward, a general trust model based on the discussed game theory approaches and scenarios is introduced to take into account the variability and features of the attack types. Consequently, we would utilize this model to any network environment (cooperative/non-cooperative game with internal/external attack). Finally, we present some applicable future trends for the interested researchers, showing the capability of facing intelligent attacks [33,34,35,36] using the evolutionary game approach [37,38,39].

The rest of the paper is structured as follows. Section 2 provides a brief overview about game theory. Section 3 outlines game theory classifications, addresses the different game types that are involved in WSN security, and provides the security properties needed for WSN security. Section 4 illustrates the proposed taxonomy of game theory defense strategies for WSN security showing the types of attacks and the types of suitable games to mitigate these attacks. Section 5 presents the proposed general trust model based on the discussed game theory types and attack types in WSNs. In Section 6, the applicable future trends are listed. Section 7 concludes the paper.

## 2. Game Theory: A Brief Overview

Game theory is an advanced branch of intelligent optimization. The model of game theory represents a game between player groups that choose to behave cooperatively or non-cooperatively and try to promote their benefits (payoffs) through the used strategy(ies) executed through the cumulative players actions. [19,20] survey the fundamental definitions of game parameters, which can be summarized as follows:

**Definition** **1.**  *A game is a description of the strategic interaction between opposing, or cooperating, interests where the constraints and payoff for actions are taken into consideration.*

**Definition** **2.**  *A player is a basic entity in a game, which is involved in the game with a finite set of players denoted by N that is responsible for taking rational actions denoted by $A_{i}$, for each player i. A player can represent a person, machine, or group of people within a game.*

**Definition** **3.**  *The Utility/Payoff is the positive or negative reward to a player for a given action within the game denoted by $u_{i}:A\to R$, which measures the outcome for player i determined by the actions of all players $A=\times_{i\in N}A_{i}$, where the symbol × denotes Cartesian product.*

**Definition** **4.**  *A strategy is a plan of action within the game that a given player can adopt during game play denoted by a strategic game $\langle N,\left(A\right),\left(u_{i}\right)\rangle$.*

In the security field, game theory application is not only limited to counteracting the effect of external intruders; it can be used to detect the malicious nodes and reveal the nodes that behave selfishly and overburden the whole network. Generally, Nash equilibrium (NE) is the intelligent solution for the social problems that has become a promising concept for wireless networks and more specifically for WSN security [19,20,21,29,40,41].

**Definition** **5.**  Nash equilibrium is a profile of optimal actions, $a^{*}\in A$, such that any player i∈N cannot benefit due to unilaterally deviating from its strategy and choosing another action [21,41]. This can be translated in terms of the utility function as, $u_{i}\left(a_{i}^{*},a_{-i}^{*}\right)\geq u_{i}\left(a_{i},a_{-i}^{*}\right)$ for all $a_{i}\in A$, where $a_{i}$ denotes the strategy of player i and $a_{-i}$ denotes the strategies of all players other than player i [19].

In Section 3, an overview of the different game types is briefly introduced, focusing on the games that can be particularly useful to describe the security situation in WSN.

## 3. Games Theory for WSNs Security

**Figure 1:**
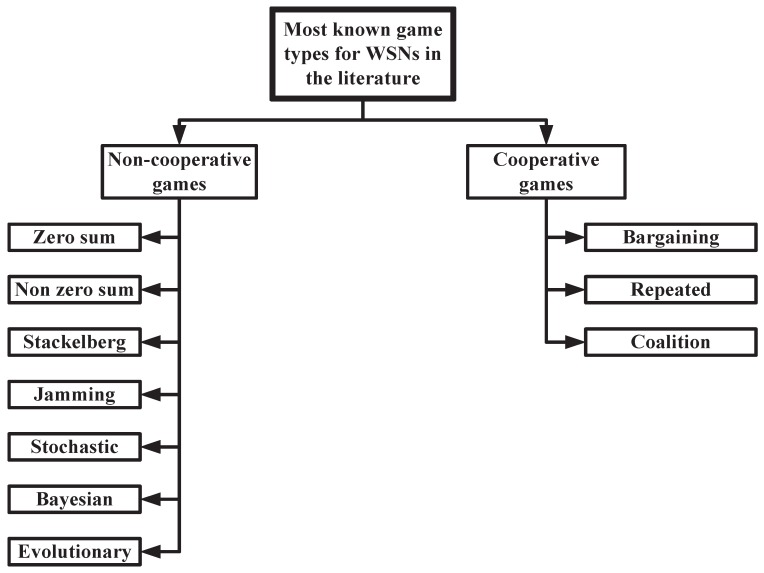
Cooperative and Non-cooperative Game Classification for Addressing WSN Security Issues.

The different game types that are commonly used to model WSNs security issues can be classified to cooperative games and non-cooperative games as shown in Figure 1. The cooperative games are represented by cooperating nodes aiming at maximizing the whole networks security against different security threats. On the contrary, the non-cooperative games involve the conflicting individual actions for which every node aims at maximizing its own payoff that opposes the others’ outcomes. Figure 1 lists the different types of games that have been used to model security problems in WSNs (Figure 1 does not present a classification for games in general, but presents the games that have been used in the literature to model WSNs security problems). In this section, a brief description of the different involved games for mitigating WSNs security threats is provided. Then, the WSN security requirements are discussed.

### 3.1. Cooperative Games

The common types of cooperative games that are used to resolve different WSN security issues are presented as follows.

#### 3.1.1. Bargaining Game

The Bargaining game represents a problem between two competitors (agents) who should cooperate; in other words, the bargaining or Nash bargaining game is modeled based on the bargaining interaction concept between two players, who request a fraction of the same benefits [21,42,43,44,45]. It can be used to model resource allocation in wireless communication networks, in which the agents aim to exploit the same spectrum, which should be fairly allocated. In Nash bargaining game, if the total requests by the two players are greater than the available resources, both requests are discarded. Conversely, if their requests are less than the available resources, both requests are accomplished. Pareto-inefficient is a result of non-cooperating players, which is solved by a Nash bargaining solution [46].

#### 3.1.2. Repeated Game

The repeated game is fundamentally considered as an interaction between two individual players who repeatedly play the game [19,21,47,48,49]. This game is also known as iterated game [47] which consists of some repetitive stages. Each stage has two players at which the current action is taken into consideration in the subsequent actions of the other players. The repeated games can be classified into two categories: finitely repeated games and infinitely repeated games [47]. In the finitely repeated games, the time period is fixed and thoroughly known. This category has a drastic defect which permits the player to act selfishly and NE equals the minmax payoff. Consequently, the punishment is not sufficient in this case. The infinitely repeated game represents the most popular notion in which the game is probably played for infinite times. The punishment is defined in this case as reducing the payoff that the non-cooperating player earns based on his reputation. The reputation is computed based on the players’ interactions.

#### 3.1.3. Coalition Game

The coalition game is a result of cooperation among a set of players acting as one player against the others aiming at maximizing the mutual outcome [21,28,50,51]. This coalition mutual benefit called coalition value. Coalition games are classified into two forms, namely, strategic form and partition form [28]. In the former case, the coalition value depends on the number of participant players in the coalition regardless of their network establishment. Conversely, in partition form, the establishment represents the intrinsic role for the coalition value [28].

### 3.2. Non-Cooperative Games

The common types of non-cooperative games that are used to mitigate different WSN security problems are presented as follows.

#### 3.2.1. Zero-Sum Game

The zero-sum game is one of the types of non-cooperative games between two players. One player is considered a maximizer that strives to maximize its gain while the other is considered to be the minimizer that aims to minimize its losses [52,53]. Consequently, it seems as a two-side conflict game or a one-side win game, at which the total utility/payoff of both players remains constant during the course of the game, $\sum_{i=1}^{2}u_{i}\left(s\right)=0$∀s∈S, where s is a strategy profile [21]. Apparently, **constant-sum game** could be transformed to an equivalent zero-sum game; and zero-sum game is a special case of constant-sum game given that the players add up their gains or losses to a constant value for any strategy profile [21].

#### 3.2.2. Nonzero-Sum Game

Nonzero-sum game is played between two or more players where the sum of players’ utilities is not constant during the course of the game [19,54]. In nonzero-sum games, all players are considered maximizers or minimizers which have no constraints on the total utility as in the zero-sum game [54]. Consequently, all the participants can gain or lose together [21].

#### 3.2.3. Stackelberg Game

The Stackelberg game is used to model two competitive players [21,47]; one is a game initiator (leader) who chooses an action from a set $A_{1}$ then the second player traces the leader’s action and chooses an action from a set $A_{2}$. This scenario is widespread in securing different WSNs where the defender acts as a leader and the attacker plays the role of the follower [2,55,56,57].

#### 3.2.4. Jamming Game

The jamming game represents a scenario between the WSN defender player against the jamming attack. The attack aims to disrupt the transmitted data stream [58,59]. The jamming game is fundamentally inspired from the zero-sum game framework in which the two players aim at maximizing their conflicting utilities. Consequently, the resultant of the two utilities are zero. Interestingly, the jamming game is used to treat the complexity constraints of implanted biomedical sensors as in [60,61]. Furthermore, underwater sensor network is one of the booming applications that utilize the concept of jamming game [59].

#### 3.2.5. Stochastic Game

The stochastic game is one of dynamic games that are played in a sequence of stages [21,62]. The stage is formulated based on a probabilistic transitions by one or more players [21,62,63,64,65]. The new state of the game is random which depends on the previous players’ actions [21,62].

#### 3.2.6. Bayesian Game

The Bayesian game falls under the non-cooperative game framework, in which the players have some information shortage while executing their actions. In other words, Bayesian game could be suitable for modeling the incomplete information interactions between players. Accordingly, a player can estimate the other players’ payoffs [19,21]. Moreover, a game theoretic approach based on Bayesian game has been developed to do intrusion detecting for wireless nodes in [66].

#### 3.2.7. Evolutionary Game

The evolutionary game is fundamentally applied for the biological networks in which the players can combine pure and mixed strategies with rational behavior to enhance some population characteristics [21]. Moreover, different WSNs applications have been modeled using evolutionary game in [67,68,69,70,71,72,73,74,75].

### 3.3. Security in WSNs: Requirements

WSNs have emerged in the recent years because of their features and the applications that they can be used in. Due to these substantial reasons some requirements should be maintained for WSNs security as follows [76,77]:

- **Confidentiality:** A transmitted data to a specific sensor node must not be understood by any other node in WSNs.
- **Integrity:** The transmitted and received data must not be maliciously altered by the participant sensor nodes in WSNs.
- **Authentication:** A sensor node uses the data authentication to verify that the received data is actually sent by the claimed sender in WSNs.
- **Authorization:** The authorization is used to guarantee that the authorized sensor nodes are only able to perform certain operations in WSNs.
- **Availability:** The WSN services must be available whenever the WSN users need them.
- **Freshness:** The data produced by the WSN sensor nodes must be neoteric.
- **Forward and backward secrecy:** Forward secrecy is used to prevent a sensor node that has left a WSN from reading any future data. Backward secrecy means preventing a new comer to a WSN from reading any previous data.

## 4. Game Theory Defense Strategies Against WSN Attacks

In this section, we propose a study of the state-of-the-art on different game trust models for WSNs security. More concretely, we propose a classification of the game theory defense strategies for confronting the WSN attacks. The classification will be carried out based on the attack type, the attacked layer, the attack being passive or clear, the attack being internal or external, the attack feature, the attack consequences, the suitable defense strategy based on game theory, and the game type.

### 4.1. WSN Attack Types vs. Corresponding Layer

WSNs are suffering from several attack types that target different layers. In [33,34,76,78,79], WSN attacks are classified based on the layer attacked and the countermeasures which can be summarized as follows. The physical layer (L1) is prone to jamming and tampering attacks in which the attacks target L1 features. The data link layer (L2) attacks (e.g., sniffing, collision, exhaustion, unfairness, stealthy, etc.) aim for deteriorating the layer facilities such as media access techniques. The network layer (L3) can be affected by certain attacks such as spoofed altered relayed information, Sybil, wormhole, etc. More specifically, these attacks aim to corrupt the network layer routing protocols. Finally, the transport layer (L4) would be infected by some attacks (e.g., desynchronization, flooding, etc.) that aim at disrupting the layer functions such as the end-to-end protocols.

### 4.2. Internal and External Attacks

In this section, the WSN attacks are categorized into internal and external attack [33] based on an extensive review of the literature. Internal WSN nodes that act selfishly or maliciously are considered as internal attacks, while the external attack is performed by some external entities aiming at deteriorating the WSNs functionality.

### 4.3. Clear and Passive Attacks

We present a study of the previous work that involved the clear and passive attacks taking into consideration the role of game theory.

#### 4.3.1. Clear Attack

The clear attack is capable of disrupting the traffic stream. It can transmit, respond, modify, and/or delete specific messages inside the stream due to the manipulation that the attacker applies. This attack can impersonate one end of a conversation or a third party. Consequently, it can prevent the infected sensor node from communicating with others.

The following papers consider the clear attack types in WSNs. In [80], the authors propose a defense attack strategy which concentrates on the jamming attack. An intrusion detection system is introduced against the attacks based on the NE concept that represents a defense strategy against the most vulnerable sensor nodes [41,81]. In [2], the attack targets the data trustworthiness of the sensor network which is mitigated by a game theoretic approach to protect the sensor nodes based on a trust score of the data item observed by those nodes. Consequently, the higher the data items trust scores are, the more trustful the nodes are. In [53], a deleterious attack scenario is considered, where a node turns malicious and then drops selected packets from a traffic stream.

Interestingly, clear attacks can cause some manipulations to the whole sensor network. Therefore, a great deal of information is needed for the defense mechanisms to be able to mitigate this type of attacks. For example, a malicious sensor node has the ability to drop incoming packets or issue route error messages to misdirect the path (e.g., blackhole attack) as presented in [82]. Also, in [83], the authors propose a game-theoretic approach to confront the attack impact of dropping the passing messages within a cluster.

#### 4.3.2. Passive Attack

The passive attack has the ability to capture the transmitted traffic without disturbing the network. In other words, it eavesdrops the incoming traffic stream without modification. The inherent risk of this attack is that the attack targets the sensitive data (e.g., the encryption codes). Moreover, this attack can represent selfish sensor nodes which strive to save their power as maximum as possible. For example, the selfish nodes go to sleep mode without a permission from the network administration, whether these nodes are involved in cooperative or non-cooperative games. Furthermore, the malicious nodes that aim at saving their power consumption by ignoring packet forwarding to their neighbors are also considered passive attack.

The following papers consider different types of WSN passive attacks. In [84], a bargaining game is introduced to confront the passive attack due to the selfish behavior of some of visual event-driven sensors. This represents a highly sensitive and vulnerable application in which the selfish nodes tend to conserve their power. In addition, in [42], the authors propose Nash bargaining games, which lead to the system Pareto optimality aiming at optimizing the network reliability. In [85], the authors consider a form of passive attack where a selfish sensor saves its power by going to sleep mode without a permission, by deceiving the network intrusion detection system (IDS). In [77], a game theoretic approach is proposed to address the passive DoS attack that causes malfunction of the forwarding mechanism of sensor nodes at which the nodes agree to forward packets but fail to do so. Furthermore, in [76], a win-lose scenario is used to model the relationship between the system and the attacker using a zero-sum game to secure the forward data path in which the attack strives to drop the passing packets. In addition, a sampling mechanism, inspired from the learning automata methodology, is applied in this game-theoretic context to treat the passive attack impact based on the reward and punishment technique [86].

### 4.4. Cooperative and Non-Cooperative Games

In the following discussion, we summarize the different game defense strategies against different attack types in WSNs. A game can be chosen to be cooperative or non-cooperative game according to the attack type and the expected penalty.

#### 4.4.1. Cooperative Games

The cooperative games can be used to model strategies that require cooperation among the participant WSN nodes to combat the different attacks. The utility function in the cooperative games is commonly represented by three main parameters, namely, cooperation, reputation, and security level between the cooperating nodes. More concretely, the NE is used as a promising notion to reach the optimal solution in different WSNs [83]. Cooperative game theories for WSNs security can be summarized as follows.

- **Bargaining Game**: A bargaining game is presented in [42] through the cooperating nodes, resulting in a Nash Bargaining Solution and Reliability in Wireless Body Sensor Networks (WBSNs). This seeks to promote the cooperation between the nodes to maximize the performance in a multi-hop WBSN and hence, the utility function distinguishes the allocated bandwidths. Three main parameters should be taken into consideration: dynamic nature, quality-of-service (QoS), and fairness of resource allocation. In [44], the power allocation problem is addressed using bargaining NE leading to cooperative relaying. The proposed model aims at achieving the optimal signal-to-noise ratio (SNR) in WSNs. In [45], the authors propose the bargaining game to attain the optimal energy management policy for a solar-powered sensor node. The proposed model obtains the optimal sleep and wake up mode for the participant sensor nodes based on the bargaining NE by treating the selfish nodes that conserve energy through blocking packets with high probability. The players’ utility function is denoted by the combination between the probability of an active mode, a sleep mode, a listen mode, a packet block, and a packet drop for the two competitive players.
- **Repeated Game**: In [49], the authors propose a game theoretic approach based on repeated game to investigate the selfish nodes throughout in CSMA/CA network. The model stimulates the selfish nodes to cooperate leading to the Pareto optimality NE. In this model, the utility function is presented as the difference between the throughput and a punishment factor of the participant nodes.
  In [85], the authors propose a specific technique controlled by the network base station to be able to detect and prevent the selfish behavior of nodes. This technique works in parallel with the Core Mesh Routing Protocol (CMP) without disrupting its functionality using a game-theoretic approach based on a repeated game to detect and prevent the selfish behavior. This game uses the power consumption as an indicator of the game utility/payoff function. Increasing the utility is considered as a positive signpost of honesty and trustworthiness and vice versa if selfish behavior is presented. Consequently, the base station investigates the utility function to ensure that whether the node is capable of going to sleep or not. The utility concentrates on the transmission cost which is based on the forwarded data. Hence, the node with the increased utility/payoff has a higher chance of going to sleep.
  In [77], a repeated game is presented targeting a defense against passive DoS attack. This model applies a defense strategy at the routing layer to promote cooperation through rewarding the cooperating nodes and accumulating reputation values over time, while punishing non-cooperative passive attackers. To reach this goal, each node plays a repeated game executing series of Nash Equilibria on a utility function that balances between cooperation gain represented by the reputation value and the cost of forwarding packets to check the routing security.
- **Coalition Game**: In [84], a defense strategy is introduced to predict the attacks and their effect using cooperating camera sensors. The model is based on a threshold for the probability of error of the captured scene. This approach provides a solution for false alarm, energy conservation, early attack prediction, and selfish behavior detection.

#### 4.4.2. Non-Cooperative Games

The non-cooperative games have been proposed to describe and model the competition between the benevolent nodes and selfish/malicious nodes. In the following we list some of the WSN security problems, which can be modeled by non-cooperative games.

- **Zero-sum Game**: In [76], a game-theoretic approach based on zero-sum game was presented seeking routing secured against external intruders. The authors have developed the utility function as a combination of the energy consumption, probability of malicious nodes, and probability of dropped packets between check nodes (one or more through the forward path) to maximize the probability of attack detection. Intrusion detection framework for smart phone systems is proposed in [41] at which two players play a non-cooperative constant-sum game with complete information to achieve NE that leads to a defense strategy for the security server. In this game, the defender wishes to enhance the security level but the attacker wants to deteriorate it. The model gathers data and checks if the security server monitors attack or not, then calculates the payoff per each case using node ID as a node identity.
  Furthermore, in [87], the authors propose an intrusion packet detection at which the classical zero-sum game is chosen as a natural model for the behavior of the attacker/defender strategies. The utility functions for both the attacker and defender take into consideration the probability of detection. In [53], a zero-sum game is proposed to mitigate the selective forwarding attack in which the infected nodes turn malicious and select some packet to drop based on a stochastic formulation. The model utility function is the difference between the defense energy budget supplied by the IDS for all participant nodes and the attack energy budget applied to turn those nodes into malicious. In [52], the authors formulate a zero-sum game aiming at maximizing the transmission capacity in underwater sensor network based on the mitigation of jammers. The utility function of this game is maximized to achieve the optimal SNR based on two observers sensor nodes against the effect of the disruption caused by the jammer.
- **Nonzero-sum Game**: In [82], a nonzero-sum game is proposed to detect the malicious nodes and their effect (e.g., dropping packets and routing error message to misdirect the path) due to the DoS attack impact. Two defense strategies are developed. The first strategy is based on dynamic source routing that aims at maximizing the payoff. The second strategy focuses on reputation and cooperation between neighbors which also aims at maximizing the payoff and enhancing the defense strategy. However, the developed algorithms suffer from unnecessary added cost in the utility function of the defender, and this cost is mainly related to continuous defending of nodes even when no attack is encountered. In [81], the authors introduce a defense strategy based on a nonzero-sum game and achieve NE using a Markov decision process to predict the most vulnerable sensor node. In [54], the proposed defense strategy aims at detecting and correcting the malicious sensor nodes that drop packets based on a nonzero-sum game using periodic collusion-resistant punishment mechanism. The model stimulates the detected malicious node(s) to react benevolently.
- **Stackelberg Game**: In [2], the attack-defense interactions are modeled using a Stackelberg game, and the NE condition is derived which is sufficient to ensure that the sensed data are truthful within a nominal error bound. The defense strategy gives an authority to the defender to be the decision maker. The model is based on a thresholding defense strategy against the attacks. The thresholding is correlated to the trust score of the data items observed by the sensor nodes to detect the effect of the followers (attacker). In [56], the authors propose a Stackelberg game for detecting the reactive jamming attack in wireless networks aiming at maximizing the signal-to-interference-noise ratio (SINR). The reactive jammer seeks to inject noise like legitimate signal at receiver. The utility function is represented by the resulting SINR, while a linear power penalty is used as a punishment for jammer and legitimate nodes. In [57], a Stackelberg game is developed to confront the external attacks manipulations based on energy defense budget against the corresponding energy attack budget. The utility function represents the resultant of the defense and attack energy budgets.
- **Jamming Game**: In [58], the authors propose a jamming game that is fundamentally inspired from the zero-sum game framework due to the uniqueness of NE. More concretely, the authors study the jamming impact on the Orthogonal Frequency Division Multiplexing (OFDM) system when the jammer is situated close to the base station. The utility function is the combination of the power level of the uncontrolled environmental noise at *i*-th state, the costs of power usage for transmitter and jammer, and fading channel gains for transmitter and jammer. In [59], a jamming game is proposed to mitigate the DoS attack (jamming attack) in underwater sensor network. The legal player utility function is maximized based on a combination of its power level, SINR, and transmission costs. The game model is classified into two cases. In the former case, the game is presented as a static jamming game that owns a single NE. In the latter case, the game is represented by a dynamic jamming game which is derived based on a Markov decision process. In other work, the jamming game is used to treat the complexity constraints of implanted biomedical sensors [60,61]. In these models, the utility function is presented in two cases. In the former case, the authors study a fixed strategy in which the jammer may or may not jam the *i*-th player. In the latter case, a mixed transmission strategy is used for the *i*-th player.
- **Stochastic Game**: In [64], the authors propose a new framework for wireless nodes virtualization in which the service providers and network operators are responsible for the QoS and spectrum management, respectively. The proposed model can handle the unknown dynamics in traffic characteristics and channel conditions. In addition, the network operator is responsible for calculating the NE based on the conjectural price of the communicating nodes leading to the optimal resource management.
- **Bayesian Game**: In [66], a Bayesian game is proposed to analyze the interactions between pairs of attacking/defending nodes. The Nash equilibrium is studied for the attacker/defender game through two scenarios, namely, static and dynamic. In fact, the dynamic Bayesian model represents the more realistic approach that allows the network defender to continuously update his decision for the existing malicious nodes. Furthermore, the dynamic scenario provides energy-efficient monitoring strategies for the defender. A new approach is suggested called Bayesian hybrid detection that adopts lightweight monitoring and heavyweight monitoring mechanisms. The lightweight monitoring is utilized to estimate the adversary’s actions, while the heavyweight monitoring acts as a last resort of defense.
- **Evolutionary Game**: In [67], the authors propose a data aggregation model called evolutionary game-based data aggregation model (EGDAM) in WSNs. The model uses a weighting method based on the pixel-level fusion between homogeneous sensor nodes to adapt the unreliable information from the nodes. In [68], an evolutionary game is used to maintain the cooperation in static and mobile multi-class WSNs, with each class managed by a different authority. Two scenarios are considered for the packet forwarding. In the former scenario, the packet forwarding is between mobile classes in which the game formulation is based on iterated prisoner dilemma. In the later scenario, the packet forwarding is between spatially dispersed stationary classes.
  In [69], building reliable and survivable networks with fault tolerance is reviewed based on bio-robustness of different biological scales, i.e., gene, molecular networks, immune systems, population, and community. In [70], dynamic hybrid fault models are proposed to achieve the reliability and fault tolerance for WSNs based on the evolutionary game. The dynamic models provide real-time prediction and fault-tolerance. The dynamics of the proposed models are involved into the time-dependent failure rate and time-dependent failure modes. The utility function of the proposed evolutionary game is formulated as reliability or survivability of the WSN in which a sensor node can be sacrificed to achieve the optimal network sustainability. The same concept of dynamic hybrid fault models has been extended [74] and extensively studied in [73].
  In [72], the WSN sensors are mapped as a biological population using evolutionary game in which the payoff function is represented by reliability of players. The dragonfly adult concept has been studied in [75] to guarantee optimal channel time sharing in WSNs using evolutionary game. Consequently, selfish behavior of nodes can be treated. The utility function is called fitness function that is represented by the sensors’ targets. In [71], the authors propose a defense strategy for WSNs aiming at reaching a stable state between the defender and the attacker using evolutionary game. More specifically, the sensor nodes can be active and dynamic to adjust their defense strategy. The payoff function is represented by a combination of some parameters of defender and attacker, e.g., reward of successive forwarded packets, security measuring cost, successful attack, failure attack, attacking cost, and probability of successful attack attempts.

### 4.5. Defense Strategies Classification

Table 1 proposes a classification that addresses the WSN attacks [33] based on an extensive review of the state-of-the-art game-theoretic approaches to deal with WSNs security. In Table 1, the first column lists the WSN attacks. In the first row, we categorize these attacks based on the attacked layer [76] (i.e., physical layer (L1) attacks, data link layer (L2) attacks, network layer (L3) attacks, and transport layer (L4) attacks); this classification takes into account both the features and consequences of those attacks. Afterward, the attack is classified as internal or external; the internal attack (In) represents an internal node acting selfishly or turning malicious, while the external attack (Ex) is caused by an external attacker. Then, we pinpoint whether the attack is passive (P) or clear/active (Clr). Further, suitable defense strategy (suitable game) is presented that mitigates the attack manipulations based on both the attack type and games features as proposed in the literature. Finally, the game type is identified.

## 5. Game Theory-Based General Trust Model

**Figure 2:**
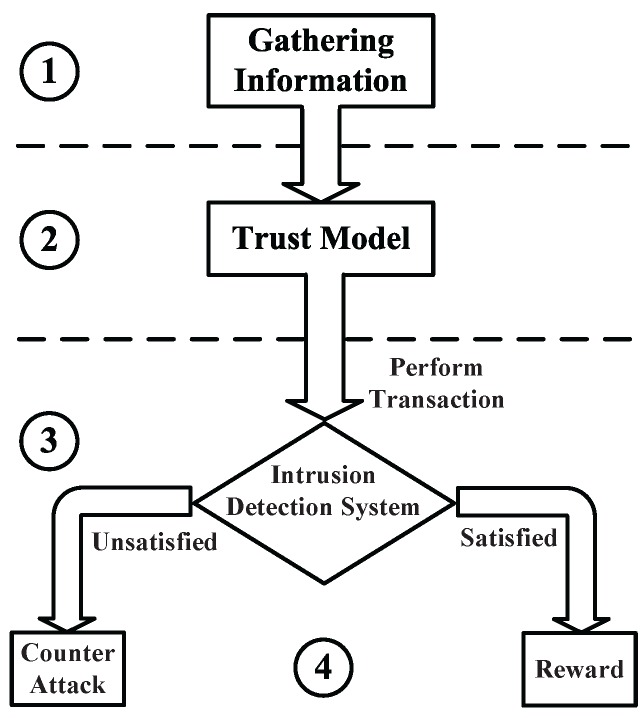
General Trust and Reputation Model Mechanisms.

The trustworthiness mechanisms are considered the foremost concern of the WSNs security specialists. Therefore, different network security frameworks are discussed in the literature [3,4,33,115]. Figure 2 shows the general process of a trust model, similar to the model considered in [116]. This trust model is divided into four main stages. The first stage involves gathering the information from the traffic stream, then followed by the second stage that applies the suitable trust model. Afterward, the analyzed data throughout the trust model is checked using the intrusion detection system within the third stage. The fourth stage is in charge of punishing or rewarding the infected or benevolent packets, respectively. This general mechanism aims at attaining energy efficient networks against the intrusion impact using the general principle of learning automata by sampling the incoming packets [86,117]. The same principle can be used to enhance WSNs security using game theory [87].

In designing the game model, the nature of WSN should be taken into account. Maintaining the same data transfer from multiple nodes, keeping low power consumption, accommodating large number of nodes, and providing timely decision are of paramount importance to the WSN operation. In fact, the reputation is the essential factor that the different game theoretic approaches rely on to establish robust trust models against the WSN threats scenarios. In the first scenario, the model targets the selfish nodes. In the second scenario, the model addresses the malicious nodes that are considered a harm for the WSN. Finally, in the most harmful scenario, the WSN suffers from selfish and malicious nodes for which an intelligent model is desired.

**Figure 3:**
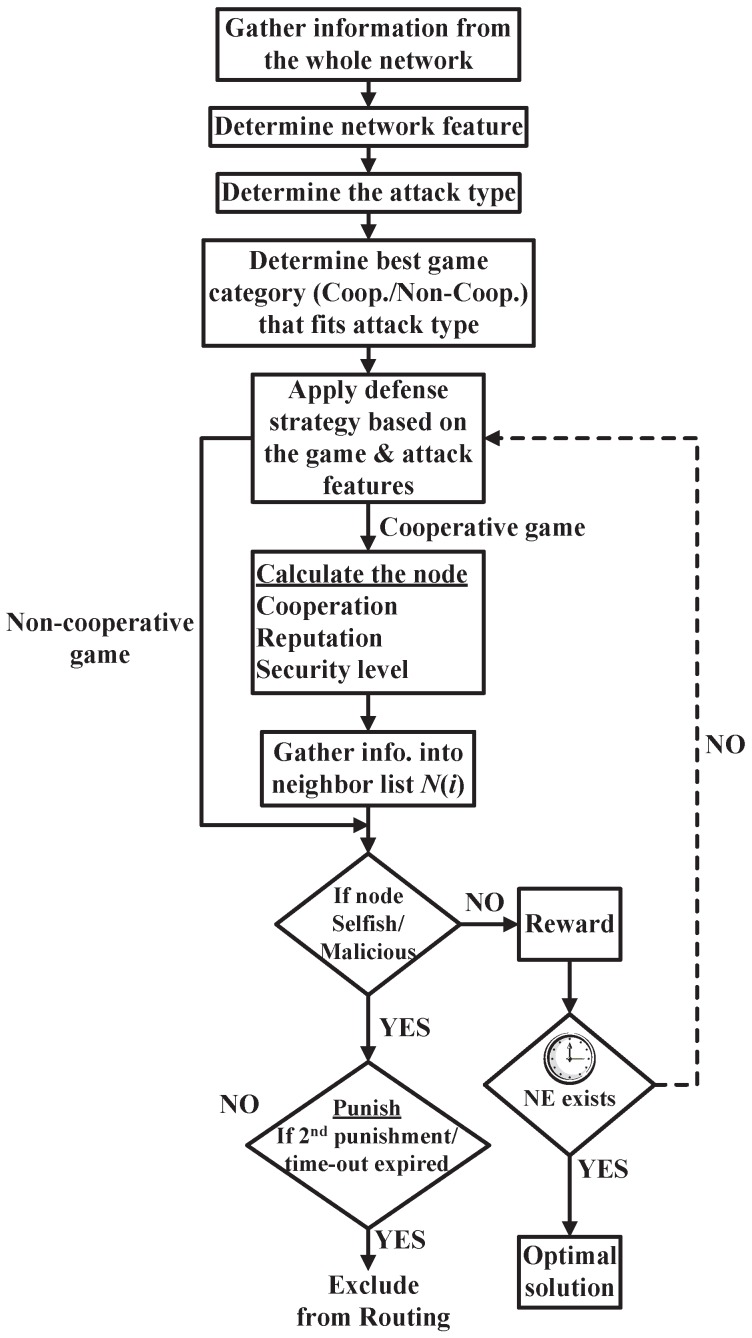
Proposed Game Theory Trust Model.

In this paper, we discuss a general trust model based on game theory to mitigate the WSNs security threats leading to detecting the malicious nodes and those nodes that act selfishly. Figure 3 illustrates the data flow of the general trust model in which the selected game is used to face the designated attack based on the observed information from the whole network. Apparently, this general system collects the needed information to select the best game that fits the detected attack and the mentioned classifications in Section 4. After this, the captured data are analyzed to pinpoint the network features, attack type, and appropriate defense strategy that will be applied, whether in a cooperative or non-cooperative game.

It can be obviously seen that two possible directions are available to apply the game-theory-based defense strategy. The cooperative process is shown as follows: the cooperating WSN nodes calculate three main parameters, namely, cooperation, reputation, and security level for every participant node. Then, these parameters are listed in an information list N(i). Afterward, every node is checked if it acts selfishly or maliciously. Consequently, the node is rewarded or punished. On the one hand, if the node is rewarded (benevolent node), the NE existence is checked. A timer is used as a threshold for the NE existence to reduce the risk in the real-time sensitive applications. This time out is application dependent. Then, if NE exists, the optimal solution is achieved; otherwise, the node will be applied again to the suitable defense strategy based on game theory. On the other hand, if the node is selfish or malicious, it will be exposed to a punishment check. Consequently, if it is the first time the node acts selfishly or due to a hardware malfunction reason, a time out will be given to the node then the node status will return again to the neighbor list N(i). Finally, if the node recursively behaves selfishly, the node turns malicious, or the time out expires, the node will be excluded from routing. The above is executed till reaching the NE based on the application sensitivity leading to the optimal solution.

The non-cooperative stages are presented starting with the suitable non-cooperative game against the existing attack. Apparently, the same steps are executed as in the cooperative process but starting from the check step that determines whether the node is benevolent, malicious, or even selfish. In addition, the non-cooperative process omits two steps. The first step is calculating the cooperation, reputation, and security level parameters. The second step is collecting the neighbor list. In centralized networks, the proposed general trust model could be deployed part of a software-defined networking (SDN) at the network controller. On the contrary, the model duties could be allotted to some of the cooperating nodes in decentralized networks. More concretely, the network operator, the service provider, the cloud provider, or a combination of them, based on the network communication structure and connectivity, could control this model.

## 6. Applicable Future Trends

The most known cooperative game models for WSNs security are based on centralized authentication. More specifically, these models are used to handle the traffic motion, maintain the benevolent nodes, and punish the selfish or malicious nodes using the cooperating nodes. However, as there are many benefits of using the centralized authentication, there are also some problems that may cause a risk for the network security such as the resources deterioration of the centralized security node. In [31], a solution is proposed, called group authentication, to reduce the risk on the centralized node. The proposed mechanism aims at distributing the security task among different correlated nodes. Therefore, the cooperating nodes are supposed to achieve the security targets in WSNs [32]. Consequently, many benefits can be addressed in WSNs as follows: reducing the whole network delay, increasing the throughput, enhancing the detection probability of the selfish/malicious nodes, saving the power consumption, decreasing the number of error messages, promoting the WSNs performance and robustness against the effect of attacks, etc.

Among the different game types, evolutionary game presents a valuable approach in which the players are rationally adapting their actions based on the iterative development of the game [21,37,39,118]. The evolutionary game has the ability to deal with the interaction among rational biological agents in a population. In addition, evolutionary game can be classified into static and dynamic hypotheses [21]. In the static hypothesis, the evolutionary game utilizes the evolutionarily stable strategies (ESS) [21]. In the dynamic hypothesis, the replicator dynamics have been involved to model the adaptation of the strategies of the players [21]. The evolutionary game framework has some advantages over the classical non-cooperative game such as equilibrium selection, bounded rationality, and dynamic behavior of players [21]. Consequently, we envision that evolutionary game models can be used to mitigate the intelligent attacks in WSNs leading to robust systems.

### 6.1. Evolutionary Game for WSNs Security

WSNs rational attacks [33,34] can cause a harmful problem due to the intelligent manipulations based on some unfixed strategy. Consequently, this issue needs an intelligent defense behavior to deal with this situation. The evolutionary game can intelligently use the mixed strategy against the intelligent attack manipulations [19,21]. In other words, the evolutionary game has the ability to change the defense strategy during the game progress against malicious attacks in WSNs [37,38]. More concretely, the strategies of players are non-deterministic in which the player can select different pure strategies with a certain probability [21,48] such as the Hawk and Dove problem [21,39,118]. In addition, we can use the dynamic evolutionary game that follows the Snowdrift game [39,118,119] which proposes a different point of view away from the other biologically based games. Dynamic evolutionary game is slightly different from the Hawk and Dove game which supports both the assigned cooperator and the other players [39,118,120]. Consequently, if the evolutionary game was generally used in the general trust model in Figure 3, it would introduce an adaptable solution for the intelligent attack dilemma.

In biological terms, the biological organisms combat each other aiming at maximizing their benefit. In particular, bacteria strive to infect/control the benevolent cells [120,121]. Similarly, the intelligent attacker in WSN aims at turning the benevolent nodes to malicious or selfish nodes based on unfixed strategy as a chameleon. Consequently, WSN nodes can be modeled as biological organisms using the evolutionary game that presents a promising solution for mitigating such situations [67,68,69,70,71,72,73,74,75]. The evolutionary game features can be summarized as follows.

- The evolutionarily stable strategies (ESS) or evolutionary equilibrium assists in the NE perfection, specifically when multiple Nash equilibria exist.
- The evolutionary game modeling is suitable for human scenarios with agents that may not have hyper-rational or strong rational behavior.
- The evolutionary game is based on an evolutionary process due to its dynamic nature, which organizes the dynamics of interactions among agents in the population. In other words, the strategy adaptation continues over time. On the contrary, most of the traditional non-cooperative games are evolved in a static setting [21].

### 6.2. Intelligent Attacks

For researchers who are interested in the recent intelligent WSNs attack types [33,34,35,36], these attacks and the corresponding features would be listed in Table 2.

### 6.3. Attractive WSNs Applications

In this section, we list some of the applicable recent trends of WSNs for the interested researchers. These applications can be listed as cognitive radio sensor networks (CRSNs), wireless underground or underwater sensor networks (i.e., under water sensor networks, Earthquake observation, agriculture applications, military uses, etc.), smart grid networks, power grid networks, energy harvesting, wireless body area networks (WBAN), and cyber physical systems (CPSs). The above applications present some security threats and game-theory-based approaches can prove to be beneficial to address these threats.

#### 6.3.1. Cognitive Radio Sensor Networks (CRSNs)

In [122], recent applications of the CRSNs have been studied in which the different schemes in CRSNs can be classified into centralized, distributed, and cluster-based. More specifically, performance enhancement is the main concern (i.e., interference avoidance, throughput maximization, QoS assurance, fairness and priority consideration, etc.). The papers that concentrate on the WSN security aspects would be summarized as follows. In [123], the authors use the interference of secondary users (SUs) to improve the secrecy capacity of the primary user (PU) in CRSN. In [124], the CR security threats have been reviewed and are classified into two main categories, namely, cognitive capability, and reconfigurability. The SU is used as a relay to improve the PHY security by which PU can enhance the secrecy rate based on game theory [125]. In [126], a sequential detection mechanism is proposed in which the SU is sequentially computing the likelihood ratio to determine whether or not to stop listening. A Stackelberg game is formulated aiming at maximizing energy saving in CR networks taking into consideration the users’ selfishness and intellectualism in [127]. In [128], two cooperation schemes are proposed to secure the PU transmission. In the former scheme, the PU selects two individual SUs to cooperate with, called relay-jammer scheme. One of the SUs acts as a relay and the other is a friendly jammer. In the later scheme, the PU cooperates with a cluster of sensor nodes whereas the scheme is called cluster-beamforming.

#### 6.3.2. Wireless Underground or Underwater Sensor Networks

The applications wireless underground sensor networks include acoustic signals such as Earthquake disaster observation, underwater sensor networks (UWSNs), agriculture applications, military uses, etc [129,130,131]. In [52], a Bayesian zero-sum game approach is proposed to confront the jammers intervention in order to disrupt the acoustic signals of UWSN. The model aims at maximizing the channel underwater capacity in the presence of different types of noise and jammer. In [59], a jamming game is proposed to model the interactions between jammers and underwater sensors. More specifically, the model concentrates on the reactive jammers that determine their jamming power based on the ongoing sensors’ traffic stream. This jammer type is more severe than responsive jammer (i.e., constant jammers). In other words, the model proposes a learning-based anti-jamming enhancement method whereas every sensor decides its own transmit power regardless of the channel gain of the jammers.

#### 6.3.3. Smart Grid Networks

Recently, smart grid networks have been combined with cognitive radio (CR) networks. In [132], the authors propose a trustful system for energy management in smart home, called smart home energy management (SHEM) to enhance delay sensitive data transmissions. The system uses the CR technology due to the promising features as reliable opportunistic data transmissions and strategic decision making. The proposed model is examined by being applied to model spectrum sensing data falsification (SSDF) attack behaviors.

#### 6.3.4. Energy Harvesting

Energy harvesting is a very booming research area in WSNs because of the limited energy nature of sensor nodes [133]. In [134], a distributed MAC protocol is proposed, called Self-Learning Energy Harvesting and Spectrum Access in Cognitive Radio Sensor Networks (S-LEARN) that combines the WSN sensor nodes and the CR technology. The proposed protocol allows the sensor nodes in CRSN to get the necessary power to transmit data packets from the small amount of power the nodes can harvest wirelessly from the environment. In [135], the random behavior of energy harvesting and energy consumption in dense small cell networks have been mathematically modeled and analyzed based on a probabilistic framework. In addition, a bandit-theoretical formulation has been developed for the cooperating users when no information is supported. Bandit is a class of online optimization problems in which no prior information is provided to an agent [135]. The agent selects a finite set of arms in successive trials and every arm produces some reward. The agent captures only the reward of the played arm and not the other arms’ rewards. Bandit is categorized based on the generated rewards of arms, e.g., adversarial bandits, stochastic bandits, etc. A problem occurs due to the tradeoff between taking actions that yield immediate large rewards and taking actions that might result in larger reward only in the future. A solution is proposed called policy or allocation rule that pinpoints which arm should be employed at successive rounds [135].

#### 6.3.5. Wireless Body Area Networks (WBANs)

Wireless body area networks are of the essential real time applications. More concretely, WBAN is used to monitor human being health (i.e., temperature, blood pressure, ECG, life activity, etc.) [42]. In [136], a game theoretic approach has been proposed to resolve Socially-aware Interference Mitigation (SIM) issue in Body-to-Body networks (BBNs). The model is classified into two main channel allocation mechanisms. In the former mechanism, the BBN stage for inter-WBANs’ communications is addressed. In the latter mechanism, WBAN stage for intra-WBAN communications is considered. Moreover, in [137], transmission scheduling is studied throughout a large number of gateways connected to one base station of medical centers taking into account the QoS requirements for the different gateways. In addition, a game theoretic approach is proposed to guarantee optimal strategy between the competitive gateways that can lead to an efficient Wardrop equilibrium.

#### 6.3.6. Cyber Physical Systems (CPSs)

Cyber Physical Systems represent the new generation of complex sensor networks that combine physical subsystems and cyber components [25,138]. Recently, CPSs have been evolved in most of control systems that rely on feedback such as power networks, social networks, smart transportation systems, sensor networks, smart buildings, etc. In [138], several future directions for sensor networks have been studied that serve CPSs based on two baselines, namely, security and privacy. Jamming attack is one of the treats of CPSs that has been mitigated by game theory models [80]. In [25], the strategic interactions between an attacker and a defender are studied using game theory in which both cyber and physical components are considered. The authors have developed and validated this model with UltraScience Net infrastructure, which was built to support high-performance network experiments. Interestingly, CPSs could be developed to combat the homeland security issues [22,23,139] based on different authorities combination to support extensive analysis leading to a robust system.

## 7. Conclusions

In this paper, we have addressed the important and challenging problem of assuring trustworthiness of sensor data in the presence of both selfish behavior and malicious adversaries in WSNs. We have carried out an extensive overview of the state-of-the-art of many game theoretic approaches that are utilized to design defense strategies to protect sensor nodes from attacks and to guarantee a high level of trustworthiness for sensed data. We have presented a classification for the different attack types, based on their features, and we have presented the different types of games that have been used in the literature to mitigate these attacks. We have also presented some potential future, active research trends for using game theory.

In addition, we have presented the concept of evolutionary game using a group policy/authentication to resist the intelligent attacks which do not use pure strategies. We expect astounding results after applying this model, such as reducing the number of dropped packets, promoting the efficiency, increasing the security level, managing the interactions between nodes, rapid attack detection, regenerating (based on the evolutionary nature of the game) and intelligent strategies against new manipulations of attacks, with the aim of producing powerful trust model based on game theory.

## Figures and Tables

**Table 1:** WSN attack classifications and defense strategies based on game theory.

Attack Type	Attacked Layer	P/Clr Attack	Ex/In Attack	Attack Feature	Attack Consequences	Defense Strategy	Game Type
Jamming [55,56,59,88,89,90]	L1 [76,78,79]	Clr	Ex	Interfere with radio frequencies	Disrupt whole/portion of the network	Stackelberg [55,56]/Repeated [88]/Evolutionary [89]/Jamming [59,90]	NC/C/NC/NC
Tampering [91]	L1 [76,78,79]	Clr	Ex/In	Extract cryptographic keys	Create/replace existing node	Repeated [91]	C
Sniffing [92,93,94]	L2 [95]	Clr	Ex	Overhear essential data from neighboring nodes	Penetration network secrecy	Stackelberg [92]/ZS [93,94]	NC
Collisions [89]	L2 [76,78,79]	P	In	Simultaneous transmission on the same frequency	Change in portion of data, Checksum mismatch at receiver	Evolutionary [89]	NC
Exhaustion [96]	L2 [76,78,79]	Clr	In/Ex	Naive implementation may continuously attempt to retransmit the corrupted packets	Resource exhaustion	Repeated [96]	C
Unfairness [42,97]	L2 [78,79]	P	In	Considered weak DoS, Intermittent exploiting the resources	Miss transmission deadline for other nodes in a real-time MAC protocol	Repeated [97]/Bargaining [42]	C
Stealthy [98,99]	L2 [78]	Clr	In	Compromise a node and inject false data through that node.	Make the network accept false data	ZS [98]/NZS [99]	NC
Energy Drain [76,98]	L2 [78]	Clr	In/Ex	Request from neighboring node to respond after massive traffic transmission	Paralyze the whole network	ZS [76,98]	NC
Conflicting behavior [100,101,102]	L3 [103]	Clr	In	Perform differently with different nodes	Decrease trust value of nodes by conflicting their reputation	Repeated [100,101,102]	C
Blackhole [82,104]	L3 [103]	Clr	In/Ex	Attract the whole traffic to be routed through it by advertising itself as the shortest route and drop all received message	Block the traffic to the sink, Expand crisis by easily combining with other extra attacker	Repeated [82,104]	C
Spoofed, altered, replayed information [66,89]	L3 [76,78,79]	Clr	In/Ex	Disrupt the network traffic	Create routing loop, Extend or shorten source route, Error message generation	Bayesian [66] Evolutionary [89]	NC
Selective forwarding [53,76,87,89]	L3 [76,78,79]	Clr	In/Ex	Transmit certain packets and drop others	A malfunction occurs in transmission process	NZS [53]/ZS [76,87]/Evolutionary [89]	NC
Sinkhole [82,105]	L3 [76,78,79]	Clr	In	Compromise a node and then attract the surrounding nodes to use it as next node	Lose huge number of packets, Retransmit lost packets, Increase delay, Exhaust nodes	NZS [82]/Evolutionary [105]	NC
Sybil [66,89,106]	L3 [76,78,79]	Clr	In	One node presents more than one ID	Exhaust nodes’ power	Stochastic [106]/Bayesian [66]/Evolutionary [89]	NC
Wormhole [82]	L3 [76,78,79]	Clr	In	Low-latency that links between two portions of the network where the messages replayed	Paralyze the whole network, Network traffic jamming	NZS [82]	NC
Hello flood	L3 [76,78,79,107]	Clr	Ex	Use high powered- transmitter to deceive neighbors that it has the trajectory towards the base station	The neighbor believe that attacker, Control the data flow	ZS ${}^{*}$/NZS ${}^{*}$	NC
Acknowledgment spoofing [66,89]	L3 [76,78,79]	Clr	In	Spoof the ACKs of overhead packets destined for neighboring nodes in order to provide false information to those neighboring nodes	Disrupt and confuse routing mechanism	Stochastic [66]/Evolutionary [89]	NC
Badmouthing [108]	L3 [109]	Clr	In	Propagate negative reputation information about good nodes	Block valid path by confusing reputation system	Repeated [108]	C
Goodmouthing (opposite Badmouthing behavior)	L3 [109]	Clr	In	Propagate positive reputation information about bad nodes	Block valid path by confusing reputation system	Repeated ${}^{*}$	C
Whitewashing	L3 [109]	Clr	In	Re-enter the network with new ID and fresh reputation	deteriorate the defense of reputation mechanism	Evolutionary ${}^{*}$	NC
Flooding [77,110,111]	L4 [76,78,79]	Clr	Ex	An attacker may repeatedly make new connection requests until the resources required by each connection are exhausted or reach a maximum limit	System traffic congestion, Cause channel capacity deterioration	Repeated [77,110,111]	C
Desynchronization	L4 [76,78,79]	Clr	Ex	Refers to the disruption of an existing connection	Repeatedly spoof messages to an end host, causing that host to request the retransmission of missed frames	Repeated ${}^{*}$	C
Intelligent behavior [112]	-	Clr	In	Selectively provide services good or bad, high or low values of recommendation according to threshold of trust rating	Disrupt trust system order indistinguishably, Increase the cost of reputation evaluation	Stochastic [112]	NC
DoS [77,82,104,110,113,114]	L1-L4 [76,78]	Clr	In/Ex	Prevent any part of WSNs from functioning correctly or in a timely manner	Split the network grid and take control of part of the network by inserting a new sink node jam and tampering network	Repeated [77,104,110]/NZS [82,113,114]	C/NC
ON-OFF	-	Clr	In	Behave well or badly by exploiting the dynamic properties of trust through time-domain inconsistent behaviors	Remain undetected while causing damage	Evolutionary ${}^{*}$	NC

Clr... Clear, P... Passive, ZS... Zero-sum, NZS... Non Zero-sum, C... Cooperative, NC... Non-cooperative, In... Internal, Ex... External; * ⋯ based on the attack and game features.

**Table 2:** WSN intelligent attacks.

Attack Type	Features
Badmouthing attack	Grants negative feedback on a node in order to disrupt its reputation.
Goodmouthing attack	Grants positive feedback about a malicious entity.
ON-OFF attack	Occurs when an adversary attempts to initiate a security attack or a mixture of attacks based irregular manner in order to make its reputation acceptable.
Sybil attack	Occurs when a node in a network claims multiple identities.
Whitewashing attack	Exists when an attacker resets a poor reputation by re-entering the system with a new identity.
Stealthy attack	Operates quietly, hides the evidence of its actions, disrupts the traffic stream from arriving the destination through malicious behavior at third party node.
Conflicting behavior attack	Deteriorates the reputation of good nodes by performing differently for different peers.
Intelligent behavior attack	Uses different behaviors based on unfixed strategy to manipulate good nodes.

## Abbreviations

The following abbreviations are used in this manuscript:

BBNs: Body-to-Body networks
C: Cooperative
CPS: Cyber Physical System
CR: Cognitive Radio
CRSN: Cognitive Radio Sensor Network
CSMA/CA: Carrier Sense Multiple Access/Collision Avoidance
CMP: Core Mesh Routing Protocol
Clr: Clear
DoS: Denial of Service
ESS: Evolutionary Stable Strategies
Ex: External
EGDAM: Evolutionary Game based Data Aggregation
In: Internal
IDS: Intrusion Detection System
NE: Nash Equilibrium
NZS: Nonzero-sum
NC: Non cooperative
OFDM: Orthogonal Frequency Division Multiplexing
PU: Primary User
P: Passive
QoS: Quality of Service
SDN: Software-defined Networking
SHEM: Smart Home Energy Management
SIM: Socially-aware Interference Mitigation
SINR: Signal to Interference Noise Ratio
S-LEARN: Self-Learning
SNR: Signal to Noise Ratio
SSDF: Spectrum Sensing Data Falsification
SU: Secondary User
UWSNs: Underwater Sensor Networks
WBAN: Wireless Body Area Network
WSNs: Wireless Sensor Networks
ZS: Zero-sum
